# Controlling unwanted memories: A conceptual review grounded in the process model of emotion regulation

**DOI:** 10.3758/s13423-025-02745-y

**Published:** 2025-08-28

**Authors:** Agnieszka Bachfischer, Irina M. Harris

**Affiliations:** School of Psychology, The University of Sydney, Brennan MacCallum Building A18, Sydney, NSW 2006 Australia

**Keywords:** Memory control, Emotional memories, Emotion regulation

## Abstract

Autobiographical memories are a crucial source of emotional states in our daily lives. While remembering negative events in the past is important to guide future behaviours and steer us away from harm, being reminded of unpleasant events too often or too intensely can have a serious impact on our wellbeing. A solution that may reconcile these positive and negative effects of negative memories is *memory control*. Being able to control when, how, and which memories to remember, based on our current goals, is similar to being able to control our emotions, which taps into the well-established field of *emotion regulation* (ER) where the ER Process Model (Gross, *Journal of Personality and Social Psychology,* *74*(1), 224–237 1998b, *Psychological Inquiry,* *26*(1), 1–26 2015) has been extensively used as a theoretical framework. The memory control field is missing such an overarching model that would provide a guiding framework and new insights for emotional memory control research and practice. In this conceptual review, we bring together three lines of well-established research – on Emotion Regulation, Involuntary Autobiographical Memories, and Memory Control – to demonstrate how the Process Model of ER can be applied to memories. The application of the ER model to emotional memories enhances conceptual clarity of the field of memory control, helps to organise existing findings, reveals meaningful similarities and differences between various memory control strategies, identifies the most potentially effective strategies, and points to the most promising future research directions.

## Introduction

Our everyday emotional states can frequently be impacted by unpleasant, upsetting, embarrassing, painful, or in other ways negative and unwanted memories. While remembering negative events in the past is important to guide future behaviours, being reminded of unpleasant events too often, too intensely, too randomly, or when trying to focus on something else can have a negative effect on people’s quality of life. Memory of emotional events (or at least their central detail) is enhanced compared to neutral events (Buchanan & Lovallo, 2001; Sharot et al., 2004), and so apart from immediate emotional experiences, such events continue to impact us further when they are evoked as memories, becoming a crucial source of emotional states in our daily lives (Engen & Anderson, 2018). Indeed, studies often employ autobiographical memory recall as a tool for mood induction (Lench et al., 2011). Having control over retrieval of unwanted memories can help preserve our emotional state, protect our sense of self, and let us focus on the current task without unwanted distractions (Anderson & Hanslmayr, 2014). Memories that cause discomfort can affect our wellbeing on a daily basis (Gamboa et al., 2017); this may be especially relevant for individuals whose minds tend to frequently wander to negative memories, which has been demonstrated, for example, in people high on general negative affectivity and in those with low attentional control (Finnbogadóttir & Berntsen, 2013). Negative memory bias has additionally been found among patients with various disorders, including depression, anxiety, attention-deficit/hyperactivity disorder, and autism spectrum disorder (Duyser et al., 2020). Some people may also be especially affected by negative memories; for example, dysphoric (del Palacio-Gonzalez et al., 2017) and socially anxious individuals (del Palacio-Gonzalez & Berntsen, 2020) showed a heightened emotional response to negative autobiographical memories. In essence, our ability to stop retrieving unwanted emotional memories when advantageous may be a major determinant of psychological wellbeing (Fawcett & Hulbert, 2020; Nishiyama & Saito, 2022).

Despite their potentially negative impact, autobiographical memories (memories of personal events) are an essential part of human lives, serving a number of important functions. Their real-life adaptive significance includes self-related, social, and directive functions (Bluck, 2003). In other words, memories help maintain continuity of the self and preserve self-concept; strengthen social bonds through providing topics for shared communications and experiences; and, finally, guide us in making plans and decisions in the present and the future. If our goal was to simply forget negative memories to feel better, this would likely impact all these functions. Negative memories protect us from potentially harmful experiences and are important to keep us and others safe (Samide & Ritchey, 2021; Schlagman & Kvavilashvili, 2008). According to Pillemer (2003), memories influence future wellbeing as persistent reminders of what is worth pursuing and what is better avoided. Knowing what to avoid would not be possible without negative memories.

Clearly then, trying to forget negative (non-traumatic) memories would negate their crucial functional and adaptive value. At the same time, however, such memories can have a serious impact on our emotional states and wellbeing. A solution that may be able to reconcile these positive and negative effects of emotional memories is memory control or regulation. Being able to control our memories to regulate their emotional impact is similar to being able to control situations, attention or cognitions to regulate emotions, which taps into the well-established field of *emotion regulation* (ER).

### Emotion regulation and memory control

ER refers to the activation of a goal to modify the unfolding emotional response, in either its magnitude or duration (Gross, 2013, 2015). It is based on the idea that, just like with negative memories, emotions have crucial adaptive functions, guiding our behaviour, but it does not mean that emotional responses are always appropriate and desirable, especially because of how much our environments have changed since those in the past that shaped our emotions (Gross, 1999). Emotion regulation, therefore, is not about getting rid of emotions – instead, it is about having control over when, how and which emotions to feel to serve our goals, cultivating emotions that are helpful and managing those that are harmful (Gross, 1998b, 2013). Similarly, we should be able to control when, how and which emotional memories to remember, based on our current goals. Emotion regulation has been shown to be crucial for people’s psychological wellbeing; this may extend to emotional memory control as well (Engen & Anderson, 2018).

To clarify terminology, most work on managing emotions has referred to *emotion regulation* (e.g., Gross, 1998b), while *memory control* has traditionally been referred to in studies on managing memories (e.g., Engen & Anderson, 2018). In biological literature, control refers to the ability to direct or command a situation or a process, while regulation refers to the mechanism or process that allows control to be achieved (Sauro, 2017). The meanings seem similar enough and the difference not consequential, and, therefore, for historical reasons we will keep referring to emotion *regulation* and memory *control.* To further clarify the scope of this paper, its focus is on dealing with negative and unwanted but non-traumatic memories; dealing with traumatic memories requires a separate line of research (e.g., Ehlers, 2010), beyond everyday-life negative memories that have been studied with memory control strategies.

The foundational work in emotion regulation came from James Gross’s (1998a, b) seminal research over 25 years ago. Since then, there have been thousands of studies on ER, with most of this work building on Gross’s (1998b) Process Model of Emotion Regulation. The model has helped to organise numerous insights on strategies to regulate emotional situations we encounter in our daily lives (see, e.g., Gross, 2015, and Webb et al., 2012, for comprehensive reviews). The memory control field, on the other hand, is missing such an overarching conceptual framework. Many studies have offered valuable insights, but they mostly focus on one or a few specific aspects or strategies. There are useful theories as well, but, again, they are relevant to specific memory control strategies only, such as the Reflection and Evaluation Model (Markman & McMullen, 2003) applicable to counterfactual strategies, or inhibitory theory of forgetting (Anderson, 2003) applicable to thought suppression. An overarching model, however, could help organise, relate and integrate the existing separate strategies and findings, providing a guiding framework and new insights for memory control research and practice. For example, by using the model to group various memory control strategies, we can theorise (and then empirically test) which strategies may lead to similar outcomes or be similarly cognitively taxing. Such a model can help find interconnections among seemingly diverse processes (Gross, 1998b) and “simplify a complex problem space” (Gross, 2015, p. 7).

**Fig. 1:**
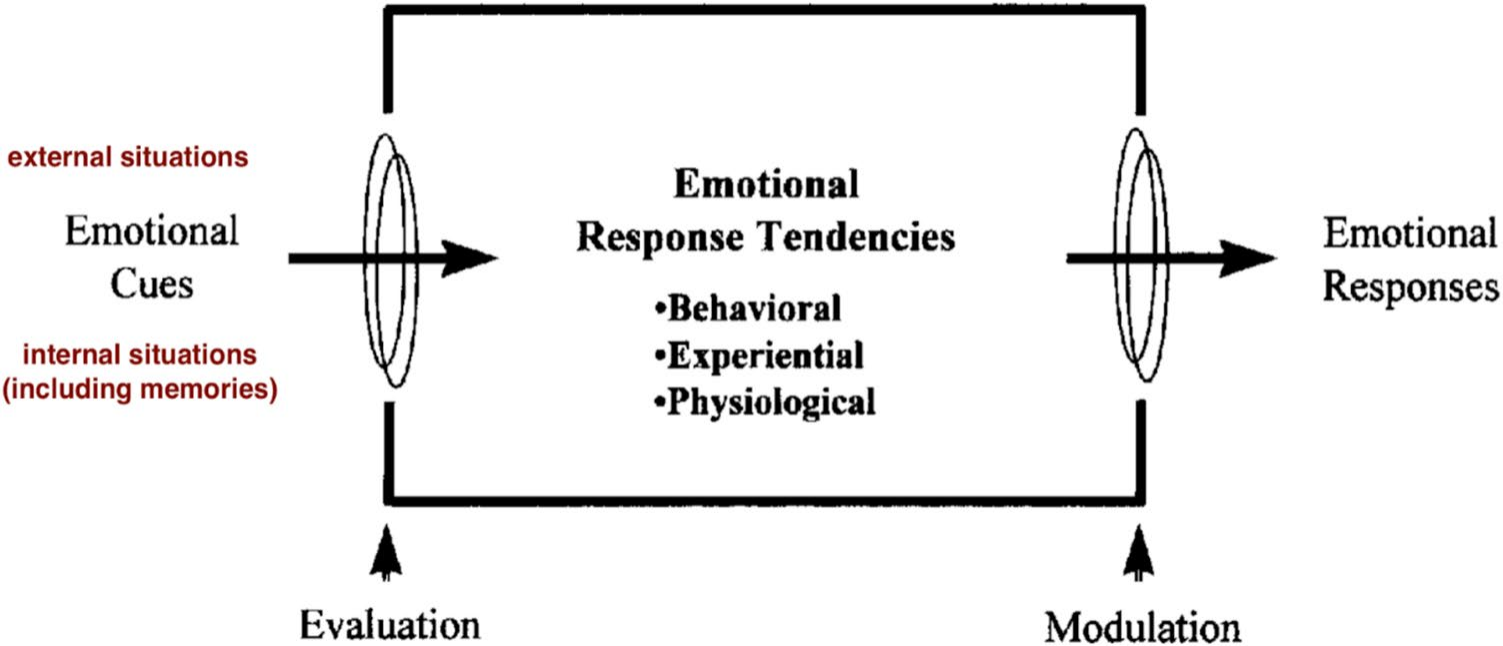
An adaptation of Gross’s emotion generation model (1998b) with both external and internal stimuli (situations)

The Process Model of Emotion Regulation (Gross, 1998b) seems very suitable as a basis for a new memory control framework. Emotions are defined as response tendencies that unfold over a relatively short time (seconds to minutes) and include changes in subjective experience, behaviour, and physiology (Gross, 1998b, 2015). The emotion of anger, for example, involves changes in posture, tone of voice, facial expressions, experience, and autonomic responding (Gross, 1998b). They last shorter than moods, and, unlike moods that are more diffuse, are elicited by specific events (Gross, 2015). Emotion regulation is defined as the activation of a goal to modify the unfolding emotional response (Gross, 2015). Such emotional responses are always in response to emotional stimuli (Gross, 1998a) or, in other words, psychologically relevant situations (Gross, 2015). What is crucial here is that such emotional stimuli or situations can be both external and internal (Gross, 1998a, 2015). Memories are an instance of such internal stimuli (along with present or future thinking) that trigger emotional response tendencies (see Fig. 1). This suggests that memory-based emotion regulation may be a subset of emotion regulation, but one with unique issues relevant to internal memories only. This paper is, therefore, an attempt to apply and adapt the well-established model of emotion regulation to control of emotional memories.

Emotion regulation research never claimed to focus on external situations only. Gross (1998a, 2015) mentions internal stimuli a number of times, for example when referring to both “external or internal emotion cues” (Gross, 1998a, p. 225) or when remarking that emotional situations may refer to either features of the external environment or “the activation of internal representations” (Gross, 2015, p. 4). Nevertheless, most theoretical and empirical articles on ER focus only on external stimuli in examples used by authors and in experimental procedures. Examples provided to illustrate ER include having a fight with a co-worker, receiving an offensive remark, watching graphic news footage, attending admissions interview, hearing loud music next door in the middle of the night, encountering an erratic driver on the road, failing an exam, or dealing with an upset child (Gross, 1998a, b, 2002, 2013). Experimental stimuli used in ER research are mostly external situations as well. They include watching a film depicting an arm amputation (Gross, 2001), watching a film with a confession of an affair (Gross, 2002), or participants describing in a diary how they emotionally regulated the most positive and negative event of the day (English et al., 2017).

In short, although internal memories evoke a whole range of emotions in our everyday lives as well, they are rarely considered in this line of research. Receiving an offensive remark, failing an exam, or having a fight with a co-worker may not only influence our emotions in the present moment, but also every time this situation is remembered in the future, resulting in a similar emotional response. The fact that the ER Process Model (Gross, 1998b) is meant for emotional responses to both external and internal stimuli, but most ER research has focused on external stimuli only, makes this paper’s focus on regulation of memories as internal stimuli especially worthwhile. It will both extend ER theory to such internal stimuli (potentially encouraging more ER researchers to explicitly consider internally triggered emotions in their work too), and, at the same time, offer novel theoretical and practical understanding of managing emotional memories grounded in the well-established ER theory and research base.

As the paper will demonstrate, some unique characteristics of internal stimuli will require re-evaluation of traditional emotion regulation strategies that are used with external triggers. As one example, one can easily avert one’s gaze away from an upsetting image (external stimulus), but it may be more difficult to get rid of a recalled mental image (internal stimulus). As another example, one can decide to walk away from a very unpleasant interaction with a co-worker, but it may be harder to later stop unpleasant memories of this interaction from popping up in one’s mind. The opposite may be true as well, with some strategies being potentially easier to apply to emotions triggered by memories; temporal distancing may be easier with memories compared to current external situations since the former are already further away in the past.

Our aim in this paper, therefore, is to demonstrate how Gross’s (1998b, 2015) Process Model of Emotion Regulation and the wealth of insights it has brought about can be applied to internal memories as opposed to external situations. This conceptual review brings together three lines of well-established research – that on Emotion Regulation, Involuntary Autobiographical Memories, and Memory Control. It shows that strategies used for emotion regulation of internal memories can be similar to those used in emotion regulation during external situations but need to be adapted to account for the unique nature of involuntary memories and their particular features. In so doing, it demonstrates that Gross’s model can be very usefully applied to internal memories as well but with many important differences, and that this approach offers helpful practical insights and future research suggestions.

The review is structured around the stages of the ER Process Model (Gross, 1998b, 2015). Each section briefly introduces each stage before suggesting potential points of difference between how this stage could apply to internal memories as opposed to external situations. It then summarises the main findings from studies that used this stage’s strategies to control memories. As is revealed in those sections, however, most studies of memory control have been based on memories of word lists or images (including sometimes emotional words or images), but few have used autobiographical memories that are the focus of this paper. Autobiographical memories are different from memories of words and pictures since they are complex, vivid, highly emotional, personally relevant, salient, highly organised in an associative network, and integrated with our concept of self (Barnier et al., 2007; Conway, 2001; Joslyn & Oakes, 2005; Noreen & MacLeod, 2013; Somos et al., 2022). Therefore, the main findings of studies that used memories of words and pictures are only briefly summarised in so far as they demonstrate specific paradigms that have been successfully used to control memories, and, therefore, have potential for control of autobiographical memories. These summaries focus on the way the paradigms have been used and on their effects, providing an important foundation that can inform studies of autobiographical memories. The scarcer studies that used various strategies specifically with autobiographical memories are then discussed in detail, including both behavioural and neuroimaging studies.

### Involuntary memories as stimuli in emotion regulation

The unwanted emotional memories that we refer to in this paper are *involuntary* autobiographical memories, as opposed to *voluntary* autobiographical memories. The latter are what we intentionally invoke, searching for some specific memory when, for example, deciding on whether to accept an offer to work with someone and intentionally remembering past interactions with them. Voluntary memories can affect our emotional states as well, but given that such memories are intentional, even if their content is unpleasant, they are already regulated or controlled by their very nature and the way they are deliberately invoked. They are, therefore, not the unwanted internal stimuli that require the engagement of regulation strategies to modify the emotional experience that this paper is concerned with.

Involuntary memories are very different; they come with more immediate emotional reactions and more mood impact than voluntary memories (Rubin et al., 2008). Berntsen et al. (2013) showed significantly shorter retrieval times for involuntary recollection, suggesting it is an automatic process with little executive control. From an evolutionary perspective for memories to have a guiding function in helping us navigate our daily lives, they would need to be mostly automatic (Pillemer, 2003). They need to be evoked quickly in new situations, without deliberate and time-consuming searching, providing us with the potentially most relevant information (Rasmussen & Berntsen, 2009). Strong emotional reactions in response to involuntary memories may reflect their automatic and unpredictable onset which leaves us unprepared to control an emotional response until it has fully unfolded (Rasmussen & Berntsen, 2009).

To sum up, involuntary memories are prevalent and inevitable, and often come with strong and immediate emotional reactions (Mace & Kruchten, 2022; Rasmussen & Berntsen, 2009). In addition, we may remain unaware for a while that our mind has wandered towards the memory and affected our emotional state as the very fact that we are mind wandering commonly stays out of our meta-awareness (Schooler, 2002). Clearly, a better understanding of how we could regulate the impact of such involuntary emotional memories is necessary.

### Related work

Such linking of memory and emotion regulation has been the focus of few discussions in the literature. Some studies linked memories and ER but in an opposite direction. For example, Öner and Gülgöz (2018) looked at how invoking a positive memory may be used as an ER strategy; Nourkova and Gofman (2022) confirmed this mood-repair effect of deliberate positive recall but also showed that even negative memories are sometimes used to help boost the mood by comparison. These studies are important but outside the scope of this paper as they do not focus on regulation of emotional responses to involuntary memories, but rather on using voluntary memories to regulate emotions.

Another attempt to link memories with ER was by Colombo et al. (2021), who investigated how using ER strategies at the time of a negative event occurrence can influence subsequent memories 1 week and 1 month after the event. Richards and Gross (2000) looked at whether using ER strategies during an event impairs subsequent memory for the event. These studies, though otherwise important, are again outside the scope of this paper, which does not focus on how to influence future memories of current events but rather on how to regulate already accumulated memories.

More recently, Luo et al. (2024) also called for further research into the interplay between ER and memory control. However, unlike our paper that conceptualises memories as emotional stimuli that can be regulated using adapted forms of traditional ER, Luo et al. treat ER and memory control as interacting but distinct processes, each capable of supporting the other. In, addition, they identify thought inhibition as the core mechanism underlying both, whereas in our framework, inhibition is one of several other regulatory processes.

More relevant to this project are discussions by some researchers who expressed similar sentiments to ours, including Holland and Kensinger (2013), who also argued the importance of studying regulation of emotions elicited by memories in addition to those elicited by external situations; these authors, however, then focused on only one ER strategy, cognitive reappraisal, and are mentioned in the relevant section of this paper. Engen and Anderson’s (2018) theoretical account also provides support for the importance of the current paper; the authors similarly stressed the importance of looking at not only external stimuli or external emotional stressors (exogenously-elicited emotions) but also self-generated emotional states (endogenously-elicited emotions); they consider memory “an equally great – perhaps even greater – source of emotional states in our daily lives” (para. 1) and memory control – a fundamental process of ER. These researchers did not attempt to apply the ER model to memory control, but their article provides a number of important insights on some attentional strategies that are useful in the relevant section of this paper.

### Process Model of Emotion Regulation

**Fig. 2:**
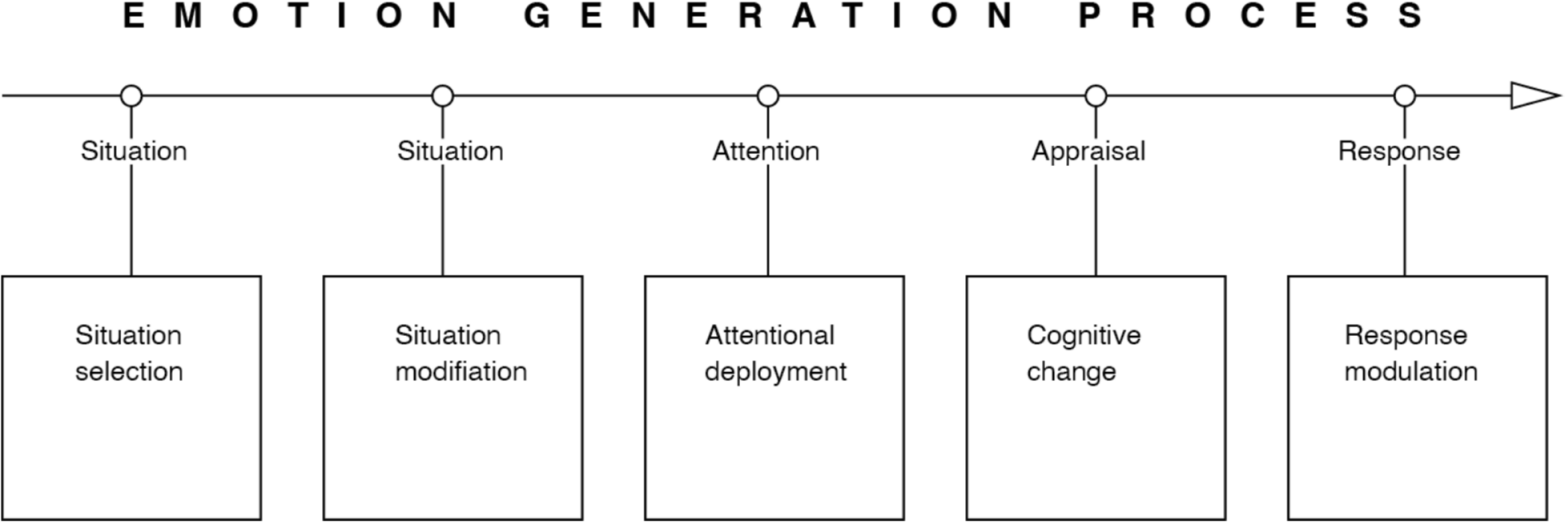
Gross’s Process Model of Emotion Regulation (1998a, b)

The Process Model of Emotion Regulation was proposed by Gross in 1998 as an analytical framework to help navigate the emerging disorganised literature and the potentially overwhelming variety of forms of ER (Gross, 1998a, b). The model sees each step in the emotion-generative process as a potential target for regulation, with five points in time at which individuals can regulate their emotions. Each of these points (see Fig. 2) represents a family of emotion regulation processes: situation selection, situation modification, attentional deployment, cognitive change, and response modulation. Movement from the first to last point represents movement through time within a given emotion-generative cycle. Different ER strategies will have different consequences in different contexts (Gross, 2013). While emotion generation is more cyclical and dynamic in nature (e.g., an emotional response such as crying or yelling creates a new emotional situation), which is acknowledged in Gross’s (2015) spiral diagrams, here we use a simplified linear diagram (Gross, 1998b) so that memory control strategies can be mapped onto it in the final section without losing clarity and this paper’s focus.

More recently, Gross (2015) proposed the Extended Process Model of Emotion Regulation. The extended model starts with the idea that emotions are generated based on a valuation system (a stimulus is evaluated negatively or positively). Such valuations involve the juxtaposition of a current representation of the world with a representation of a desired state of the world. A negative evaluation of the stimulus may generate, for example, anger. This emotion-generating system is referred to as a first-level valuation system. There is, however, another valuation system (a second-level valuation system) that takes the emotion-generating first-level system as a target and, in turn, evaluates its activity either negatively or positively (e.g., evaluating unfolding anger as not helpful in the situation). Based on its valuation, this second-level system then activates actions to modify the emotion-generation activity in the first-level valuation system (e.g., attempting distraction to reduce the anger). The first-level valuation system, therefore, refers to emotion generation, and the second-level valuation system to emotion regulation.

These valuation systems are interacting during three chronological stages of the emotion regulation cycle (Gross, 2015). These stages are the identification stage (evaluating whether to regulate emotion), the selection stage (deciding what strategy to use to regulate emotion), and the implementation stage (implementing a suitable tactic). Each stage represents a potential point of failure in emotion regulation (Gross, 2015).

The following sections apply the ER Process Model (Gross, 1998b, 2015) specifically to memory-based emotion regulation as opposed to external-situation-based emotion regulation which it has commonly been used for in research, moving through the five chronological stages of emotion generation (that are potential targets of emotion regulation), and then through the three chronological stages of emotion regulation.

## Applying the Process Model of Emotion Regulation to involuntary emotional memories

### Situation selection/modification

Gross (1998a, 1999, 2015) defines *situation selection* as taking actions to find oneself in a situation likely to lead to desirable emotions. In other words, it refers to approaching or avoiding certain people or situations based on their likely emotional impact. Examples of situation selection would be avoiding interactions with an unpleasant co-worker or deciding to go to a party or skip it. *Situation modification*, on the other hand, is defined as changing aspects of the situation one is already in, modifying the local environment to alter its emotional impact and make it more likely to lead to desirable emotions; examples include putting away a rejection letter or taking out a board game for children to play during a boring dinner (Gross, 1998b, 2015).

Both *situation selection* and *situation modification* refer to shaping situations in a physical environment, both based on changing the external world to which one is exposed (Gross, 1999, 2015). Gross (1998b, 2015) acknowledged that changing a situation may mean creating – selecting – a new situation and so it may be difficult to always draw a line between the two; situation selection and modification, therefore, are considered together in this paper as well.

Applying situation selection and modification to memory-based emotion regulation would mean choosing certain situations or changing aspects of these situations so that they are not likely to evoke unwanted emotional memories. Here it is useful to draw on many excellent insights offered from research on involuntary memories that tell us in what kinds of situations such memories are actually evoked or triggered.

Pioneering diary research into involuntary memories by Berntsen (1996) showed that such involuntary memories were very common and had identifiable and clear cues (in 93% of 700 memories) that were either aspects of the surroundings or aspects of the current cognitive activity that matched part of the remembered event. Common cues included objects, activities, people, places, generic and life themes, wordings, feelings, and sensory experiences, emerging from the everyday environment of individuals. Further analysis of the diary studies (Berntsen, 1998) revealed that the majority of the involuntary memories arose when participants’ attention was diffuse. Mace (2004) established that abstract cues (thought or linguistically based) accounted for 68% of all involuntary memory cues (with only 3% sensory cues, such as tastes or smells). Mazzoni et al. (2014) similarly found that abstract verbal cues (labels such as *dog*, *ball* or *cake*) were most effective in evoking involuntary memories. Faber and D’Mello (2018) revealed that mind wandering is more likely to consist of memory retrievals (as opposed to future thinking) when people engage in semantically rich activities such as reading or watching television. The most important findings from these studies, therefore, are that involuntary memories are triggered frequently and rapidly by a large number of clear cues that include not only situations that match the evoked memory through exact details, but, more commonly, through much broader semantic/linguistic concepts.

These findings have important ramifications when looking for potential situation selection and modification strategies to regulate involuntary memories: there seems to be no way to be able to select or modify a situation in a physical environment to make it less likely to evoke unwanted memories. Cues are so prevalent, so accidental, and so quickly and automatically matched when we are not even paying attention, that there does not seem to be any way to ever predict and influence them in our physical environments. They could be in any conversation we have, in any passage we read, in any song we hear, or in any cognitive activity we carry out – in every aspect of the semantically rich world around us.

This seems quite different from typical ER discussions where external situations can be more easily predicted and influenced – for example, we can avoid interacting with someone but cannot avoid cues that may trigger memories of past interactions with them. Any concept, object, place or wording encountered anywhere can unexpectedly trigger the memory. Clearly, regulation of emotional memories seems much more difficult than that of external emotional situations if we cannot use the first two stages of the ER model. Regulating emotional responses is more demanding and cognitively costly the further we are in the process of them unfolding, or, in other words, the further we move along the Process Model stages (John & Gross, 2004).

This has motivated us to look for ways the environment *could* be modified to make it less likely that unwanted memories will be evoked. While, as shown above, it seems impossible to directly influence the situation in a physical environment where cues are ubiquitous and outside our control, there could be a way to influence, to a degree, whether these cues end up evoking specific unwanted memories. In other words, if there could be a way to change parts of our mind’s associative network of cues and memories, certain memories would be less likely to ever be evoked regardless of whether the cue is encountered.

The reason why these strategies would be placed in these first stages of the model (that traditionally refer to modifying situations in physical environments) is that they could prevent some unwanted memories from being evoked, which means that there would be no need to move to the following stages (*attentional deployment* or *cognitive change*), just like modifying a situation stops an unwanted emotion from ever being generated. Changing cue-memory associations does not change the external environment, but the internal landscape of our associative network. For Gross (1998a), the first two stages of the model refer to external environments only, but then again, only external situations have been extensively used in ER research. The focus of these two stages is on selecting or modifying the stimulus in an environment – in externally based ER, it would be an external environment, but in internally based ER, it could be an internal one.

Another reason why changing the associative network of cues and memories cannot be placed in further stages of the model (which traditionally refer to internal environments) is that the next stage, *attentional deployment*, is about something else: choosing whether or not to focus on the stimulus when the memory has already popped up in one’s mind. Changing the internal landscape of one’s associative network of cues and memories to avoid certain memories to even be evoked is very different, and more equivalent to changing external environments to avoid negative emotional situations from even happening (as in traditional ER research).

Ways to modify the internal associative network of memories would be based on important discoveries about how a given cue isolates a specific memory (Berntsen et al., 2013). Involuntary memory is due to spreading of activation within an associative network, from the cue representation to related concepts in the autobiographical memory system (Berntsen, 2009; Mace, 2007; Schlagman & Kvavilashvili, 2008). Retrieval of such memories is due to some degree of overlap between the cue and some distinctive feature of the memory content (Berntsen, 1996, 2009; Schlagman et al., 2009). This is different from voluntary retrieval from autobiographical memory (Conway & Pleydell-Pearce, 2000) where cues initiate a top-down hierarchical search, starting with life themes, moving on to general and then specific events.

#### Cue-overload principle/retrieval-induced forgetting

An important finding that could be explored here is the *cue-overload principle* (Berntsten, 2009; Watkins & Watkins, 1975), which states that “the probability of recalling an item declines with the number of items subsumed by its functional retrieval cue” (Watkins & Watkins, p. 442). According to Berntsen (2009), involuntary retrieval depends on the number of memories associated with each cue; cues associated with many memories are less likely to evoke involuntary memories than cues associated with few memories. Thus, the likelihood of a cue triggering a specific memory depends on the extent to which the cue is uniquely associated with the memory. If cue-memory associations could be manipulated in the mind’s associative network in advance, specific memories (such as the most undesirable ones) could be less likely to be involuntarily evoked in our subsequent interactions with the world. Berntsen et al. (2013) demonstrated the effect of manipulating the number of cues associated with stimuli during encoding, finding that cues associated with a small number of stimuli were indeed more likely to elicit involuntary memories than cues associated with many stimuli. If an unwanted autobiographical memory cue could be manipulated by deliberately coming up with a number of other (more positive) memories that can be associated with the same cue, it may stop evoking the unwanted memory every time the cue is encountered. Phrases or words would likely be effective as such cues (Mace, 2004; Mazzoni et al., 2014).

Another paradigm relevant to manipulating the internal associative memory network is Retrieval-Induced Forgetting (RIF), which is based on the idea that repeated retrieval of a given memory will strengthen that memory but impair access to other memories that share the retrieval cue (Anderson et al., 1994). In this paradigm, participants first study a series of category-exemplar pairs, such as Fruit-Orange (Anderson et al., 1994). The exemplars of a given category, therefore, share the category label as a retrieval cue. Participants then engage in directed retrieval practice on half of the items from half of the categories (these items being termed *Rp+*) where they are presented with a category name together with an exemplar stem (e.g., Fruit-Or…). Afterwards, there is a final recall test where participants are cued with each category name and asked to freely recall any exemplars of that category. Retrieval-induced forgetting is demonstrated by impaired final recall of the unpractised items from the practised categories (e.g., Fruit-Strawberry, termed *Rp−*) compared to the final recall of items from the unpractised categories (those categories, e.g., Furniture, for which none of their exemplars had been given retrieval practice, termed *Nrp*). RIF seems to be predominantly driven by inhibitory processes that weaken competing memories that interfere with target memories during retrieval practice, but associative interference (response competition from stronger, practised items) contributes to it as well (Murayama et al., 2014; Storm & Levy, 2012). It is important to note that what is demonstrated in the experiments is impaired recall, not actual forgetting of the memory because the memory may be retrieved at a later time. However, to be consistent with the large body of research based on this paradigm, we continue to refer to it as Retrieval-Induced *Forgetting.* Similarly, in further sections of this paper, we refer to other relevant paradigms (Directed *Forgetting* and Suppression-Induced *Forgetting*) using the traditional naming as well.

The paradigm has been extensively studied with word stimuli and more recently with pictures (e.g., Kovacs & Harris, 2022; see Murayama et al., 2014, for a meta-analytic review), including emotional items (Kuhbandner et al., 2009), but, as discussed in the *Introduction*, such stimuli are very different from autobiographical memories. Still, several studies did demonstrate RIF of autobiographical memories, starting with Barnier et al. (2004) and Hauer and Wessel (2006), whose procedures were subsequently followed by other researchers (García-Bajos & Migueles, 2016; Glynn et al., 2018; Harris et al., 2010; Stone et al., 2013). All of these studies showed that autobiographical memories that were not practised but competed with practised memories through a shared category cue ended up being recalled less often than baseline memories. In Barnier et al.’s (2004) study, the degree of impairment of unpractised-related memories was 10–15% below the baseline of unpractised-unrelated ones, and 20–25% below practised memories.

The original procedure used by Barnier et al. (2004) was based on the traditional RIF paradigm of Anderson et al. (1994) but extended to autobiographical memories. Participants generated autobiographical memories in response to verbally presented emotional cue-words that were negative (e.g., *horrified*, *sickness*), neutral (e.g., *patient*, *polite*), and positive (e.g., *excitement*, *happy*), with four unique memories generated per cue. For each memory, participants gave a short verbal description to the experimenter and provided a personal word that would remind them of each memory. They then studied the category word, personal word, and autobiographical memory associations (e.g., hardworking – exams – studying for my final exams). In the retrieval practice phase, participants studied half of their associated memories for half of the categories. The experimenter presented the category-word and personal-word pairs, and participants were asked to respond to each pair with the correct autobiographical memory. In the final test, they were asked to verbally recall all the memories associated with each category word.

As pointed out by Somos et al. (2022), however, when participants retrieve fewer Rp- items (unpractised memories from the practised category), which is normally taken as the evidence of the RIF effect, it could simply mean that they could not recall which memories they reported during the elicitation stage, not that they actually forgot the memories. To overcome this limitation, Somos et al. created authentic autobiographical memories under the disguise of on-campus team-building exercises that consisted of 20 games, divided into two sets performed in two different locations. The location was then used as a category cue during selective retrieval practice 48 hours after the experience. The study demonstrated the practice effect for the practised items and the RIF effect for the Rp- items. Participants were worse at recalling taking part in unpractised-related games, which shows that what had become less accessible was their actual autobiographical memories, not which memories they provided as part of an experiment.

The RIF effect was observed for both positive and negative autobiographical memories by Barnier et al. (2004) and Stone et al. (2013) with adult participants, and also by Glynn et al. (2018) with 8- to 9-year-old children. Some researchers found the RIF effect only for negative, not positive, memories (García-Bajos & Migueles, 2016; Harris et al., 2010; Hauer & Wessel, 2006). What is important for this paper’s focus, however, is that all the researchers who used the RIF paradigm with autobiographical memories found the forgetting effect for negative memories, making it an ideal candidate as a memory control strategy for negative memories. Even though the final tests used in those studies do not elicit involuntary memories, their findings are likely to translate to involuntary memories as well. Retrieval of voluntary and involuntary memories draws on the same system of memories (Conway & Pleydell-Pearce, 2000), differing in the way cues are intentionally (in voluntary memories) or automatically (in involuntary memories) used to elicit a memory. Since the RIF studies show impaired accessibility of unpractised-related memories even when deliberately searching for memories matching the cue, such memories may be even less likely to be automatically triggered when the cue is encountered in daily life.

As a memory control strategy, therefore, if individuals were to identify unwanted memories that tend to bother them, along with cues that trigger these memories, such memories could then be assigned to a category (cue) together with other, more positive memories, and then only these positive items could be repeatedly practised, reducing the accessibility of the unwanted memory. This would extend the cue-overload principle further with the retrieval-induced forgetting (not only would there be more memories associated with a cue to decrease the likelihood of a specific unwanted memory being evoked by this cue, but this likelihood could be decreased further by practising retrieval of only the other – positive – memories associated with the cue). In addition, recall of unwanted memories could also be potentially impaired when instead of other related memories, individuals would generate imagined future scenarios matching the same cue as the original memory, as demonstrated in Ditta and Storm’s (2016) experiments; since the imagined scenarios need to differ from actual memories sharing the same cue, this may require the inhibition of related autobiographical memories, also leading to retrieval-induced forgetting.

#### Counterconditioning

A related line of research that could also be of potential interest here is counterconditioning (see, e.g., Keller et al., 2020, for a review), which is a form of inhibition that interferes with the expression of the original learned response associated with a specific stimulus (e.g., “dogs are dangerous”) through a new strong association of an opposite valence, such as, for example, that “dogs are fun”. The new association is formed through creating a competing memory (of spending fun time with a dog, in this case) against the original memory of a threatening encounter. This seems related to RIF that could involve retrieval practice of positive memories associated with the same cue as the original negative memory. In counterconditioning, however, a new experience is created to form a competing memory (an instance of fun playing with a dog), while in the RIF strategy proposed earlier, an individual would draw from already existing positive memories associated with the stimulus/cue. Counterconditioning has a different goal to that of RIF (changing the learned response to the stimulus, instead of impairing explicit recall of memories), but, as it does work through forming a new memory association, it could be relevant as another potential way of modifying an individual’s internal cue-memory network. In terms of everyday strategies of memory control, though, it would likely not be feasible to create new memories for many cues that tend to bring up upsetting memories (e.g., school camp, previous workplace), as is done with counterconditioning and often used with specific phobias; instead, individuals may be able to come up with competing positive memories to replace the negative memory association through RIF practice. Counterconditioning could perhaps still be a potential strategy, however, for those cue-memory associations where individuals are not able to recall strong positive memories associated with a specific cue, and, therefore, would benefit from creating them.

In summary, in the situational stages, regulating emotional memories seems much harder than regulating external emotional situations that can be more easily selected or modified in people’s external environments. Promising studies, however, show how memory cues could be manipulated to effectively change an individual’s ‘internal environment’ – their associative network of cues and memories – to reduce the likelihood of the most unwanted emotional memories being accidentally evoked during people’s daily lives. As such strategies would belong to the first (situational) stages of the Process Model, they would likely be effective as they act even before an emotional response starts unfolding (John & Gross, 2004). Further work is needed to empirically test the effectiveness of such strategies.

### Attentional deployment

The next stage of the ER model is *attentional deployment* (see Fig. 2), which is reached if a situation could not be selected or modified in the first stages, and a negative emotional stimulus (such as an external situation or memory) could not be avoided. According to Gross (1998b, 2015), this stage is about directing attention with the goal of influencing one’s emotional response. This includes shifting attention away from something undesirable or redirecting attention to something else. It still acts early in the emotion-generating process so it may be very effective in down-regulating negative emotions.

In external situations, one has a choice to shift attention using senses (which can be as easy as closing the eyes, looking away, or plugging the ears) or using internal focus. With memories, however, all the attentional deployment needs to happen internally. While being able to control attention internally may be much harder, there is plenty of research evidence to show that it is possible and that it may be effective. This has been shown by studies using two well-established paradigms – Directed Forgetting and Think/No-Think – as well as methods where attention is directed towards neutral aspects of a negative memory.

#### Directed forgetting

In Directed Forgetting studies (Bjork et al., 1968; MacLeod, 1998), individuals are presented with items to study (usually words or pictures), with each item followed by a direct instruction about whether it should be remembered or forgotten (*item method*). After a break, participants are asked to recall or recognise all the items – both the ones that they were asked to remember and the ones they were asked to forget. In another version of this paradigm, people are instructed to forget a previously studied item list and to learn a new list instead (*list method*), again being tested later on both lists. Studies show that people do recall and recognise items they tried to remember better than the ones they tried to forget (e.g., Gamboa et al., 2017; Wang et al., 2019). Gamboa et al. (2017) investigated strategies used by participants when following instructions to remember or forget, and found that attentional shift and attentional suppression were key in the directed forgetting process. Indeed, the preferred strategy of 94% participants in a typical directed forgetting group was relocation of attentional resources away from to-be-forgotten items towards to-be-remembered items. In addition, 78% of participants used some type of thought suppression to disregard the to-be-forgotten words. Both of these strategies may be used together in successful forgetting but, crucially, both demonstrate the successful use of attentional deployment with memories.

While this has been demonstrated in studies with memories of words or pictures, including emotional ones (Marchewka et al., 2016), only few researchers have attempted to apply the Directed Forgetting paradigm to autobiographical memories. Two such studies showed successful directed forgetting – one with autobiographical memories recorded in diaries during participants’ daily lives (Joslyn & Oakes, 2005), and one with memories generated in the lab in response to word cues (e.g., *seaside*, *train*, *hurt, unfair, cuddle*) (Barnier et al., 2007). In both studies, participants did remember fewer autobiographical memories when instructed to forget them, and such directed forgetting was successful regardless of the emotional valence of the memories. In Joslyn and Oakes’s study, those instructed to forget recalled 24% fewer events than *remember* participants in Experiment 1, and 14% fewer in Experiment 2. In Barnier et al.’s study, memories subjected to *forget* instructions were recalled at a rate of 55% across six experiments, while memories subjected to *remember* instructions were recalled at a rate of 66%.

Both of these studies used the *list* method of Directed Forgetting. In the diary study (Joslyn & Oakes, 2005), after they returned their Week 1 diaries, half of the participants were told that Week 1 events would not be part of the memory study and were advised to make an effort to forget the Week 1 events to facilitate memory for Week 2 events. The remaining participants were asked to remember all the events from both weeks. At the end of Week 2, all participants were tested on how well they remembered all events. They were also asked to explain strategies used; most participants from the *forget* group noted that they tried to forget Week 1 events by either ‘not thinking’ about them or by focusing on Week 2 events (which suggests attentional suppression and attentional shift, respectively). In the lab study (Barnier et al., 2007), after the List 1 memories were generated, half the participants were similarly told to forget the memories recalled so far. All participants were then told to generate and try to remember List 2 memories. Participants were then tested on their recall of all the memories they generated earlier, with the same level of detail.

Such list-method forgetting seems to involve control of maintenance and retrieval of memories, as opposed to item-method forgetting that seems to involve control of encoding; the list method is, therefore, more relevant to memory control of already encoded autobiographical memories, the focus of this paper. The studies reviewed above demonstrate that participants are able to control, to a degree, how much they remember from their autobiographical memories by using attentional strategies, which could decrease accessibility of unwanted memories.

#### Think/No-Think

Another paradigm that has provided evidence of attentional deployment potential in emotional memory control is Think/No-Think by Anderson and Green (2001). This paradigm, sometimes referred to as Suppression-Induced Forgetting, is similar to Directed Forgetting, but instead of participants being told to forget some items and being free to choose an attentional strategy to achieve that, in Think/No-Think they are directly instructed to stop an item from entering awareness – to suppress attention to it. Participants are first trained on unrelated word pairs and asked to remember them all. Then, during a recall test, a cue from one of the pairs appears and the participants are instructed to either recall the associated word, or to not think about it. It is emphasised that they should not allow the associated memory to enter consciousness at all, rather than just not say it aloud – the goal, therefore, is preventing the memory from entering awareness. To increase the need for such internal suppression mechanisms, participants must fixate on the cue for the entire time it is on the screen (to avoid simply looking away, which would be more external regulation).

The Think/No-Think paradigm has been used in numerous studies, yielding strong evidence that, again, not only are participants able to suppress the unwanted memories, but also that after they have managed to stop retrieval of items a number of times, they do not recall them so well later. In addition, forgetting increases with the number of times the memory is avoided (Anderson & Green, 2001). This has been shown not only with neutral word pairs, but also with emotional words (Joormann & Tran, 2009) and emotional images (Depue et al., 2007; Gagnepain et al., 2017; Küpper et al., 2014). Anderson and Green (2001) proposed that active suppression reduces the availability of the memory representation, rendering it inaccessible to subsequent retrieval, but further research revealed that direct suppression is not the only mechanism used by participants – another one is thought substitution (attentional shift) where recall of unwanted memories is avoided by recalling an alternative memory instead (Engen & Anderson, 2018). Nishiyama and Saito (2022) found that both suppression and substitution induced forgetting of emotional scenes. Neuroimaging studies indicate that the two strategies rely on different neural mechanisms (Benoit & Anderson, 2012), even though the forgetting effects are very similar. The memory suppression condition involves signals from the right dorsolateral prefrontal cortex that are believed to act on hippocampal inhibitory neurons to prevent retrieval, whereas the memory substitution condition seems to rely on left frontal mechanisms underlying selective retrieval of competing memories (Benoit & Anderson, 2012).

The research on suppression-induced forgetting has been primarily based on memories of words and images. As with retrieval-induced forgetting and directed forgetting, however, there have been some limited attempts to apply the paradigm to autobiographical memories. The first such attempts were undertaken at the same time by Noreen and MacLeod (2013) and Stephens et al. (2013). Noreen and MacLeod proposed the Autobiographical Think/No-Think procedure (ATNT), where participants generated positive and negative autobiographical memories based on cue words (e.g., *theatre, wildfire*), briefly describing each memory (with instructions for each description to include causes, consequences, and personal meaning) and a single personal word that would remind them of each memory. After 7 days, participants studied details of memories they had provided originally from transcripts of their verbatim descriptions, in association with the cue–personal word pairs. They were then instructed to recall the memory associated with some of the cue-personal word pairs presented in a green font (*think* condition), and to avoid thinking about the memory associated with other cue-word pairs presented in a red font, preventing it from coming to mind (*no-think* condition). Finally, participants were asked to recall all the autobiographical memories when cued with the cue-personal word pairs. Two ATNT experiments demonstrated forgetting effects for details of positive and negative autobiographical memories; the forgetting effect in Study 1 was about 8%, and in Study 2, 11%. The forgotten details were central to the memory (including causes and consequences of the event). Stephens et al. (2013) also asked for both positive and negative memories to be generated to cues such as *humorous* or *jealous*, along with a personal title; in the *think/no-think* phase, half the cues were presented in green (*think*), and half in red (*no-think)*. Unlike in Noreen and MacLeod’s study, in Stephens et al.’s experiments there was no learning phase, and in both the *think/no-think* phase and the final test, participants were asked to recall both the memory’s title and its short description in response to the cue (rather than being given the cue *and* the memory title as in Noreen and MacLeod’s study). There was again evidence of impaired recall of suppressed memories in terms of memory details, relative to baseline memories.

Apart from thought suppression, forgetting effects of autobiographical memory details can be achieved through thought substitution as well. Noreen et al. (2016) used the ATNT paradigm again but, apart from participants being instructed to either think about the memory or to suppress thinking about it, this time the third option included memory substitution (think of an alternative memory to suppress the original one). The alternative memories to the same cues were of the opposite valence to the original memory and had been generated and learnt earlier by the participants along with the original memories. Findings showed similar forgetting effects in both *no-think* and *substitute* conditions. In the post-experimental questionnaire, participants expressed having greater difficulty “not thinking” about the original memory in the suppression than in the substitution condition, suggesting the latter may be an easier everyday memory control strategy.

#### Other memory suppression/substitution paradigms

More evidence that people are able to suppress unwanted autobiographical memories has come from studies that focused on successful suppression without investigating the later forgetting effects mentioned above. In Hu et al.’s (2015) study, such memories were generated during a mock lab-based crime (stealing an object). Participants in the memory-suppression group were told they should never allow the memory of the mock crime to come to mind during testing, and they should not engage in distracting thoughts. Electroencephalographic (EEG) measurements showed reduced amplitudes of the P300 event-related brain potential when people were instructed to suppress the memory; as the amplitude of P300 has been linked to conscious recollection of episodic memories, the researchers suggested it as evidence of successful down-regulation of neural activity underlying retrieval of participants’ memories, making the memories less consciously accessible. Satish et al. (2022) investigated how successfully autobiographical memories could be suppressed using self-report measures – in the *no-think* condition, participants immediately after suppression attempts rated on a scale from 1 to 3 whether the memory intruded when they tried to suppress it (1 – *the memory never entered awareness*, 2 – *the memory briefly entered awareness*, 3 – *the memory was in awareness for the entire trial or repeatedly came to mind*). Morally wrong memories intruded more frequently (in 20% of trials) than morally right memories (17%), but, in addition, more memory intrusions occurred during the first (25%) than the second half (12%) of the session. The last finding suggests that repeated suppression attempts reduce intrusions and, importantly for memory control strategies, that suppressing unwanted memories can get more successful with practice and repetition.

While the studies reviewed above demonstrate that people are able to control attention to negative autobiographical memories, which can also reduce the memories’ accessibility later on, an additional potential benefit of attentional strategies is reduced negative emotions in response to these memories, which seems to be studied less often as an effect of these strategies. Satish et al. (2024) showed that substituting a negative memory with an imagined safe-space scenario (e.g., imagining being at a beach, with all the accompanying sights and sounds, such as waves crashing) can reduce negative feelings associated with memories of morally wrong actions; participants felt more positive after repeated attempts at such memory control of morally wrong memories. As the safe space was chosen by each participant and stayed the same for the duration of the experiment, this could be a feasible everyday-life strategy that does not require much effort, recalling the already imagined safe space every time one wants to use this strategy. Another strategy tested by Satish et al. was actively imagining a more positive, alternative scenario of a negative memory, which also induced a positive emotional response. Both safe-space and positive-alternative strategies also reduced feelings of guilt and shame associated with the generated morally wrong memories. In Stephens et al.’s (2013) Think/No-Think study mentioned above, suppressed negative memories not only became less detail-specific in the final test but were also rated by two individuals (who were blind to the experimental conditions) as less negative. While not using autobiographical memories, but less personally relevant memories of emotional scenes, Nishiyama and Saito (2022) showed that in Think/No-Think, direct suppression only reduced arousal without improving emotional valence in response to the aversive scenes, whereas thought substitution reduced both high arousal and negative valence. All these findings show important beneficial effects of attentional memory control on emotional states.

#### Changing focus within the memory

Other attentional strategies that focus on improving emotional response to negative memories include those that do not try to stop attention to the memory completely by suppressing it or bringing up a substitute memory, but, instead, attempt to change which aspects one focuses on within the same memory. Research has shown that individuals are able to change their attention away from arousing aspects of a negative image maintained in their working memory to neutral aspects of the same image, and that doing so reduces self-reported negative emotion and the late positive potential (LPP), an EEG measure of emotional reactivity (Thiruchselvam et al., 2012). While this has been shown not with autobiographical memories but rather with the contents of working memory straight after an image presentation, these findings could be tested with imagery brought up from autobiographical memories as well. De Brigard et al. (2019) showed that attentive remembering (actively focusing on specific details of negative memories) is the best strategy (with the largest effect size) to reduce negative affect associated with negative autobiographical memories compared to imagining a better and worse version of the remembered event, or not recalling the memory at all. As an example of attentive remembering, if a person’s memory was about missing a train, they were instructed to focus on some salient detail of the train station, such as the smell or a poster on the wall. The researchers suggested that such attentive remembering may bias people’s attention towards specific aspects of the negative event in their memory, and while they did not refer to these details as neutral (only as salient), from the example provided it seems that it was similar to Thiruchselvam et al.’s study in terms of focusing on neutral aspects of an otherwise negative memory, which led to less negative emotional rating of the memory.

#### Impact and individual differences

All the studies reviewed above are very relevant to the work on potential strategies to control emotional memories as they show that: (a) individuals are able to control attention to autobiographical memories, suppressing or substituting them, or shifting attention to certain aspects of the memory, (b) they can get better at it with practice, (c) suppressing and substituting helps reduce the accessibility of previously controlled negative memories (at least in terms of their details), and (d) such strategies can also reduce negative emotions associated with the memories and improve positive mood. The last two points may be directly related, as Stephens et al. (2013) suggested that reducing detail-specificity of memory may limit its emotional impact (at least for emotional but non-traumatic memories, such as a final conversation with a former partner or a poorly delivered talk). Noreen et al. (2016) similarly proposed that successfully suppressing details associated with an unpleasant memory may gradually weaken it and reduce some of the associated painful emotions an individual experiences (while still remembering the event itself). This could be ideal as memory control since, as discussed in the *Introduction*, the goal should not be to forget completely that events happened (to preserve the adaptive, guiding function of memory) but to reduce their negative emotional impact; the latter could be achieved through attentional strategies that make unwanted memories less accessible (and so less likely to be triggered involuntarily) but that also reduce their negative emotionality if they are ever recalled again. In addition, even without those more long-term effects, being able to stop an unwanted memory from entering awareness may provide an immediate benefit of preserving our current emotional state.

An important consideration, however, is raised by findings of another study by Noreen and MacLeod (2014), where the same participants from their earlier study (Noreen & MacLeod, 2013) – except those who could not be recruited again – were asked to take another recall test months after they engaged in the *think/no-think* session. Specifically, the delay was 12–13 months for Study 1, and 3–4 months for Study 2 of the original paper. The forgetting effect for suppressed memories did not persist following this delay. An especially important finding was that there were different results for participants who were good and poor suppressors during the original studies. For good suppressors, after the delay, there were simply no more differences in the recall of details for *think*, *no-think*, or baseline memories (no more of the forgetting effect of previously suppressed memories). However, poor suppressors could now remember *more* details of *no-think* memories. While it cannot be established from the study, the researchers proposed that failed attempts to suppress may have led to higher accessibility due to a preoccupation with that memory after noticing it was unsuccessful during the original session. Such findings provide an important reminder that individual differences in suppressing ability need to be taken into account when proposing any real-life memory control strategies. Deficits in suppressing of *no-think* memories have been demonstrated in various populations, for example, among individuals with ADHD (Depue et al., 2010). In Nishiyama and Saito’s study (2022), when using direct suppression, individuals with more severe anxious/depressive symptoms showed smaller effects on both valence and arousal reduction, but when using thought substitution, this effect was not impacted by anxious/depressive symptoms. This is further supported by findings from research on emotional nouns where only non-depressed participants demonstrated forgetting of negative words with suppression; depressed participants only forgot when guided to use earlier learnt substitutes instead (Joormann et al., 2009). Poor suppressors, therefore, may need either more practice or even a different memory control strategy altogether, possibly relying on substitution rather than direct suppression.

All in all, there is promising evidence that individuals can improve emotions associated with negative autobiographical memories by using attentional deployment strategies, although such strategies seem harder than when regulating emotions of external situations where it is possible, for example, to simply shift gaze away from a negative stimulus. Specific strategies include negative memory suppression, substituting a negative memory with a positive one or with an imagined safe space, and attending to neutral aspects of a negative memory. Further empirical research is necessary to understand these strategies better, including their possible beneficial effects and individual differences in the ability to implement them.

### Cognitive change

*Cognitive change* is the next stage in the ER Process Model (see Fig. 2 earlier), and can be used when one has not diverted or suppressed attention to an emotional stimulus. It is about evaluating the situation one is in, changing its meaning so as to alter its emotional significance, either by changing how one thinks about the situation or about one’s capacity to manage the demands of the situation. Examples include changing what a job interview means to someone (e.g., a chance to learn more about the industry), or reminding themselves that an upsetting event does not involve them directly (Gross, 1998b, 2015).

One specific form of cognitive change, cognitive reappraisal, has gained increasing research attention because of its associations with a number of positive health outcomes (Gross, 2002). Two main reappraisal strategies include *distancing* and *reinterpretation*. Distancing refers to simulating a new perspective of an original event, with a changed psychological distance, whereas reinterpretation focuses on deriving a new meaning or outcome from a situation; both have the aim of altering the emotional impact of the original situation (Powers & LaBar, 2019).

Strategies at this stage of the ER model could be applied to autobiographical memories in a similar way as to external situations. The way cognitive reappraisal is implemented does not seem to differ much for internally or externally generated emotions – by this stage, the focus is internal in all cases. For external situations (e.g., another passenger having a particularly loud and long phone conversation during a train ride), there were easier choices in the early stages of the model based on the physical environment – avoiding peak travel (situation selection), moving to a seat farther away (situation modification), or blocking the noise by using ear plugs or headphones (attentional deployment). For *a memory* of a particularly loud and long phone conversation that spoiled one’s journey, emotion regulation strategies in the early stages were much harder, as discussed above (trying to weaken the strength of associative cues of the memory so it is less likely to be evoked, and suppressing, substituting, or focusing on neutral aspects of the memory when it does pop up). At the cognitive change stage, however, the strategies seem to apply similarly to both scenarios – internally re-evaluating the meaning or distance of the emotional situation, whether it is a current event or a memory of the event. Not surprisingly, there have been a number of studies focused on cognitive reappraisal of autobiographical memories, which is different from scarce autobiographical memory studies discussed earlier in the situational and attentional stages.

Potential differences could still be at play though. On one hand, reappraisals of current situations have a chance of being made anew, while memories may already come with potentially negative appraisals consolidated with them, having been recalled together over and over again. Still, Conway (2005) suggests that the constructive nature of memory retrieval leaves it malleable and open to modulation by personally relevant goals. It may also be easier to reappraise an older situation (memory) rather than a current one as the emotional impact is already naturally more distanced and faded, and less likely to be still relevant to the current goals.

#### Reinterpretation-based reappraisals

A number of studies have demonstrated successful reappraisal – both reinterpretation and distancing – in terms of immediate effects on emotional responses to recalled memories. Regarding reinterpretation, Fabiansson et al.’s (2012) participants recalled an anger-inducing memory and were then instructed to think of the memory using rumination or cognitive reappraisal. Reappraisal (reinterpreting the event in a different, more objective and positive way) produced the lowest levels of self-reported anger. In another study, participants recalled a series of highly arousing negative autobiographical memories and then were asked to focus on them using *feel*, *accept* or *analyse* strategies, with the latter (objectively analyse the reasons underlying their feelings) designed as a cognitive reappraisal strategy (Kross et al., 2009) that led to significant drops in self-reported negative emotions.

Another way in which people can reinterpret the meaning of a memory is by using counterfactual thinking, which refers to imagining alternative ways in which the event could have occurred instead (De Brigard et al., 2019). Upward counterfactuals refer to thinking in which we imagine an alternative event better than the one in the actual memory; downward counterfactuals are the opposite, and involve imagining worse alternatives than the original memory. It seems there is an important difference in how these two kinds of counterfactuals are used to regulate emotions. If people imagine a negative memory in a more positive way, this is the same as memory substitution strategies (Satish et al., 2024) mentioned in the *Attentional deployment* section; upward counterfactuals, therefore, are an attentional, not cognitive change strategy. However, if people imagine a memory in a downward counterfactual way (even more negative than the actual memory), then just focusing attention on this worse scenario would likely not improve their negative emotions. On the other hand, if, as an extra step, they use the imagined worse scenario to compare it to what actually happened, this may lead to a change in their evaluation of the actual memory as not as bad as it could be, consequently improving their emotions (De Brigard et al., 2019). With this extra step, downward counterfactuals would be considered a cognitive change strategy – it is not just about bringing one’s attention to another version of the memory, but then also performing an additional cognitive step of reinterpretation.

A model of two modes of counterfactual thinking that supports this distinction was proposed by Markman and McMullen (2003), one referring to experiential simulation of the counterfactual as if it was true (suggesting an attentional strategy), and the other one referring to using the counterfactual as a reference point against which to evaluate one's present standing (suggesting a cognitive change strategy of reinterpretation). Importantly for negative memories control, the model proposes that only the experiential (attentional) use of *upward* counterfactuals and only the evaluative (cognitive) use of *downward* counterfactuals can lead to positive emotions (with the opposite use leading to negative emotions). Such effects were found in two experiments by McMullen (1997), where participants were coming up with downward and upward counterfactuals of autobiographical memories using either sole attentional focus (when instructed to vividly imagine the counterfactual) or using reappraisal (when instructed to evaluate the counterfactual against the factual events in the memory). Satish et al.’s (2024) study mentioned in the *Attentional deployment* section also supports the model since upward counterfactuals that led to more positive emotions were clearly used as an attentional strategy – participants had to actively focus on imagining the better scenario before moving on to the next memory, leaving no time for a cognitive reappraisal. To sum up, simply imagining upward counterfactuals (better alternatives) has been shown to be a promising attentional strategy, while imagining downward counterfactuals (worse alternatives) followed by a comparison of the actual memory to this worse alternative seems a promising cognitive reinterpretation strategy of autobiographical memory control.

#### Distance-focused reappraisals

Apart from reinterpretation, distance-focused reappraisals have been successfully applied to autobiographical memories, too. Parikh et al. (2022) showed that temporal distancing (thinking how one may feel about the event 10 years from now) was effective in reducing negative emotional rating of regretful memories. In another study (Boucher & Scoboria, 2020), participants were either asked to write about sensorial and contextual details of their autobiographical memory (designed to lead to an immersive view of the experience), or about the significance of the event within one's life (suggesting a distanced view of the experience, given the temporally extended whole-life narrative focus). The latter strategy reduced negative emotions in response to the memory more than the details-describing strategy.

Apart from temporal distance, distance-focused reappraisals can also focus on changing the perspective away from the self-engaged one to a naturally more distanced perspective of an impartial observer. Robinson and Swanson (1993) demonstrated that emotions experienced when recalling an autobiographical memory decreased when people shifted from a first-person to an observer perspective. Speed et al. (2020) developed the Autobiographical Emotion Regulation Task (AERT) where participants recalled autobiographical memories, including those that triggered intense negative emotions. They were then instructed how to engage in cognitive reappraisal of the recalled situations, and an example provided was about trying to see the event from the perspective of an impartial observer (suggesting a distance-focused reappraisal). Afterwards, participants rated their overall subjective emotion; negative emotions were reduced, compared to the control condition. Katzir and Eyal (2013) also found that asking participants to change from a first- to third-person visual perspective reduced feelings of sadness and anger. It is important to note, however, that such benefits were not replicated for feelings of shame; this was suggested to occur due to the experience of shame involving self-evaluation as well as evaluation of the self from the perspective of others, where a third-person (others’) perspective may exacerbate these emotions. Küçüktaş and St Jacques (2022) reviewed relevant findings and also found that visual perspective differentially impacts self-conscious and basic emotions. Adopting an observer-like (third-person) perspective might reduce basic emotions but amplify self-conscious emotions. An implication of these findings is that any consideration of a third-person perspective as a distancing reappraisal strategy for emotional memory control would need to carefully differentiate the kinds of emotions it is applied to, to avoid worsening negative emotions instead of improving them.

#### Long-term impact

While the studies reviewed above demonstrated an immediate reduction of negative emotions in response to memories reappraised in several different ways, some studies also looked at the impact of such reappraisals after a delay. In Holland and Kensinger’s study (2013), participants reappraised negative autobiographical memories in order to either decrease or increase their emotional reaction to them. Emotional ratings suggested that after a delay of 30 minutes, memories previously reappraised to decrease emotional reaction continued to be rated as significantly lower in emotional intensity than events that had appeared with the instruction to increase emotional reaction.

Another study that looked at impact delayed by 1 week was by Doerig et al. (2021), who investigated the effect of non-invasive transcranial direct current stimulation (tDCS) applied over the right dorsolateral prefrontal cortex (dlPFC) on reappraising a negative autobiographical memory. Participants reappraised their personal negative memory according to an audio-guided task (that instructed them, e.g., to consider that in every situation, there is also something good). Those in the reappraisal condition displayed a greater decrease in negative valence of the memory and less arousal compared to the control condition. In addition, those who reappraised under tDCS experienced their negative memory as less negative after the reappraisal than those who reappraised under sham stimulation. However, there were comparable effects on positive and negative self-reported evaluation in the reappraisal and control condition after 1 week. This may suggest that one reappraisal session, even with tDCS, is not enough to have long-term effects on negative valence of memories.

One study, on the other hand, found enhanced positive emotion in response to a negative memory even 2 months after reappraisal. Speer et al.’s (2021) participants first reactivated negative autobiographical memories (in response to a list of common life event cues, such as *family vacation*) by writing a description for each memory. Those in the experimental group were asked to elaborate on the most positive aspects of each memory (e.g., describe something you learned or something positive that occurred because of this). The experiments provided evidence that finding positive meaning led to enhanced positive emotion and more positive content at future retrieval 1 week and 2 months later, compared to the control group that was recalling the memory naturally. The findings showed that in future recollections of the original memory (1 week and 2 months later), participants included aspects of the positive elaborations from the original session, suggesting that such positive-meaning finding leads to future recollections that include components of the initial recollection and of the positive elaboration. Such memory updating was reflected in a greater hippocampal and striatal dissimilarity across retrievals in the positive-meaning-finding group compared to the natural-recollection control group, suggesting that positive elaborations change how memories are represented in the brain across time.

Such cognitive reappraisal of negative memories with long-term effects was the focus of Samide and Richey’s review (2021). They used the term *retrospective reappraisal* in relation to reappraisal of memories, as opposed to *online reappraisal* (reappraisal of ongoing experiences). In their literature review, they found that the memory mechanisms and neural processes involved in facilitating successful long-term retrospective reappraisal are largely unknown. They proposed that after the reappraisal has been completed, the memory is likely to be reconsolidated with the updated content that will be retrieved the next time the memory is recalled. The researchers hypothesised that the completeness and strength of memory reactivation before reappraisal should be related to the likelihood of successfully updating the memory, suggesting that the memory’s emotional component needs to be reinstated to be modified. The authors suggested further research is necessary to test these ideas, but if successful, retrospective reappraisal could help maintain adaptive emotional memories with their functional value but with reduced negative emotional impact.

Future studies on such long-term memory updating with positive reappraisals could draw on insights from a large body of research on memory reconsolidation. There is plenty of evidence that memories do return to a fragile state when reactivated, making them susceptible to manipulations during the time-limited reconsolidation window (Schwabe et al., 2014; Scully et al., 2017). The proposed role of reconsolidation is to allow memories to be updated so they can keep their current relevance in the face of new information, and maintain their predictive and adaptive function of guiding future behaviour (Lee, 2009). Many studies used reconsolidation theory to induce post-retrieval amnesia for previously established memories, mainly using pharmacological manipulations to interfere with reconsolidation after retrieval (see Scully et al., 2017, for a review). Within the context of everyday-life memory control, such a complete inability to retrieve negative memories does not seem beneficial due to their guiding function; more relevant are studies that focus on updating, not forgetting, a memory. Memory reconsolidation approaches could be used to effectively update a negative memory with a more positive reappraisal. In addition, research that uses behavioural, instead of pharmacological, means would be relevant to everyday-life memory control.

There have been a number of studies that focused on such modifications of memories using non-pharmacological means during the post-reactivation reconsolidation window (see Scully et al., 2017, for a meta-analytic review of episodic memory reconsolidation). Many focused on memories of words or pictures; for example, when learning sets of 20 objects in Hupbach et al.’s (2007) study, participants were either reminded of learning the first set of items or not before learning List 2 items 2 days later. Those who received a reminder incorrectly intermixed items from List 2 when recalling List 1 items another 2 days later, suggesting that reconsolidation involves incorporation of new information into the original memory. Wichert et al. (2013) found that when learning a new set of pictures immediately after reactivation of memories of pictures seen 1 week earlier, the strength of new encoding (learning new pictures three times, as opposed to learning them only once) played a critical role in updating of the original memories (reducing their accuracy due to intrusions from the newly learned pictures).

Several studies provided support for such reconsolidation-driven updating of autobiographical memories as well. St Jacques and Schacter (2013) manipulated memories of a museum visit by reactivating them first (using photographs participants took during the visit as triggers) and then showing participants photos from a different tour. At a later test, the earlier reactivation increased false recognition of photographs not experienced during the museum tour. Schwabe and Wolf (2009) had participants recall (reactivate) and write down an autobiographical memory from within the past 2 weeks, in response to a cue adjective such as *happy* or *angry*; participants in the experimental group then memorised a folk tale. A week later, all participants tried to recall as many details as possible from the same autobiographical memory they wrote about during the first session; the memory for these events was impaired for those who learnt the folk tale during the original session. This, however, was seen only for neutral, not emotional, events. Adjectives such as *angry* were again used as cues for recalling and writing down autobiographical memories in Piñeyro et al.’s (2018) study; the memories had to be a minimum of 7 days and a maximum of 5 years old. After 10 minutes (Experiment 3), participants in the experimental group were exposed to an audiovisual clip with positive images and classical music. Seven days later, and then again 30 days later, participants were asked to recall details of the same memory they recalled on the first day. The positive audiovisual experience after the initial memory reactivation decreased the number of recalled negative details of the memory both 7 and 30 days after the initial session (while neutral, non-emotional contents remained unaffected). This effect was found only if the audiovisual clip was presented during the reconsolidation window (not later than 6 hours after the initial memory reactivation). In addition, the effect was found only for women, which, as Piñeyro et al. suggested, could be due to women’s higher strength of the memory reactivation as they recalled more details of memories than men did. All these studies suggest that autobiographical memories can be updated when new information is presented during the reconsolidation window, but more research is needed to better understand the influence of factors such as the emotional valence of the memory, the strength of the memory reactivation, the strength of new encoding, and the kind of memory content affected.

Cognitive reappraisal, therefore, seems to be a promising memory control strategy, potentially improving negative emotions triggered by a memory not only immediately after reappraising, but even long term. Future research should focus on comparing the effectiveness of various cognitive reappraisal strategies, including those based on reinterpretation and distancing, extending findings on traditional reappraisal of current situations to reappraisal of emotional memories; memory reconsolidation theory and body of evidence could also inform future attempts to update negative memories long-term with positive reappraisals.

### Response modulation

*Response modulation* is the last stage of the Process Model of Emotion Regulation (see Fig. 2). It refers to directly influencing experiential, behavioural, or physiological components of the emotional response after the emotion is already well developed, modifying emotion-related experience and actions. Examples include using relaxation, physical exercise, or food to change the emotional response, or attempting to suppress the expression of emotion, such as when trying to maintain a neutral facial expression to hide emotions or stop oneself from crying (Gross, 1998a, b, 2015). Research shows that suppressing expressions of emotions actually increases the response of the sympathetic nervous system (Gross, 1998a). For participants who were asked to hide (suppress) their emotional reactions when watching a disgusting film, the experience of disgust did not decrease but their sympathetic activation increased (Gross, 2002). Compared to no regulation, expressive suppression leads to decreased positive but not negative emotion experience, greater activation in emotion-generative brain regions such as the amygdala, and less liking in social contexts (Gross, 2013, 2015).

Only a few studies investigated expressive suppression specifically in relation to emotions generated by memories. Suppression of emotional expression is likely to similarly fail to decrease negative emotional states (Gross, 1998a), as demonstrated by Dalgleish et al. (2009), who found that when participants were instructed to suppress their negative feelings when writing about a distressing memory, only individuals low on general negative affect were able to suppress their emotions, but those who scored high on general negative affect, exhibited an even greater increase in negative emotions than those in the no-instruction condition. Other, more beneficial response modulation strategies, such as relaxation or exercise (Gross, 1998b), are likely to work the same as during emotions based on external situations and, therefore, would not require a separate line of research. There does not seem to be any evidence to suggest that response modulation of emotions evoked by memories would be different from emotions evoked by external situations in terms of its immediate effects.

#### Social sharing

However, as with the previous stages, regulating emotions triggered by memories has an additional impact apart from the immediate one – it may change the memories themselves, making them less detailed or less negative, which is important for any future recalls of the same memory. One response modulation strategy, social sharing, has indeed shown such positive long-term effects. Skowronski et al. (2004) found that frequently disclosed memories and memories disclosed to many types of people (e.g., sibling, friend, teacher, stranger) showed a stronger fading of negative affect than those not disclosed often or to fewer types of people. In addition to these correlational studies, experimental manipulations (participants disclosing their memories in 3-minute conversations with other participants) also showed that social disclosure increased the fading of negative emotions associated with autobiographical memories. Muir et al. (2015) further demonstrated positive effects of social disclosure on the emotional intensity in response to unpleasant autobiographical memories, both immediately and after a 1-week delay. Private verbal disclosure did not show such effects (telling the story of the event privately, but as if telling someone else). Their study revealed the crucial role of the listener in social disclosure – sharing the memory with an interactive listener who provided verbal feedback led to the fading of emotional intensity of unpleasant memories, but talking to a non-responsive listener increased their emotional intensity. Social disclosure to interactive listeners, therefore, seems a promising strategy of memory control that can decrease emotional intensity of negative memories. Muir et al. (2015) suggested further studies could examine the effectiveness of other types of feedback as well (e.g., by email or social media).

## Applying the valuation stages of emotion regulation to emotional memories

Apart from the chronological stages of the emotion-*generation* process that were the focus of the sections above, the Extended Process Model of ER (Gross, 2015) also considers chronological stages of the emotion-*regulation* process. The identification stage is concerned with evaluations whether to regulate emotion, the selection stage – with deciding what strategy to use to regulate emotion, and the implementation stage – with implementing a suitable tactic. Each stage involves a separate valuation system that is important in successful emotion regulation (Gross, 2015). Considering these three stages in future research could help discover further insights on successful memory control strategies. There seem to be no studies yet that would specifically focus on strategies for identification, selection, or implementation of memory control, so the main focus of the sections below is to suggest relevant future research directions.

### Identification stage of emotion regulation

When regulating emotions elicited by negative memories, there are a number of challenges in the *identification stage* (deciding whether to regulate emotion). As discussed in earlier sections, involuntary memories happen mostly when our attention is diffuse; they are quickly and automatically evoked by accidental cues in the environment (Berntsen, 1998). This means that we are often unaware that our mind has wandered towards the memory and affected our emotional state, as mind wandering typically remains outside of our meta-awareness (Schooler, 2002). This may lead us to only identify the need to regulate an emotional memory late, not noticing for a while that it has brought up unpleasant emotions. This seems different to emotion regulation of current external situations where we are more likely to be aware of what is happening in the world around us and how it affects us. This represents a serious difficulty in detection of the need to use memory control. Many of the studies reviewed in earlier sections of this paper were conducted in the lab where participants were explicitly asked to control their memories – that is, the identification of the need to regulate came externally, from experimenters’ instructions. Participants were also focused on the task (at least to the extent of successfully following the instructions). A challenge in daily lives would be for individuals to make it a habit to notice by themselves when their minds wander to unwanted memories and to then attempt to regulate them (e.g., suppress or substitute thoughts, use distancing). Researchers (e.g., Zedelius et al., 2015) have proposed strategies to increase people’s meta-awareness of mind wandering, which could be potentially very helpful here. Empirical studies are needed to investigate whether individuals could get better at first noticing negative emotional memories coming to their mind and affecting their emotional states, and then attempting to immediately regulate them.

In addition, if people do notice their mind wandering to a negative memory and triggering an unwanted emotion, before they decide to use a regulation strategy, they need to be aware that emotions and memories are able to be regulated. Research shows that people who believe that emotions are controllable are more effective in controlling their emotions (Tamir et al., 2007). Training could help increase individuals’ understanding of the memory controllability and of positive consequences of emotional memory control.

### Selection stage of emotion regulation

If individuals identify a need to use emotional memory control, this brings them to the next stage of emotion regulation – the *selection stage*. At this stage, they need to decide which strategy to use – for example, suppress or substitute the memory, reinterpret its meaning, attempt to distance themselves from it, or share it with someone. People could be taught about such strategies and which of them tend to be beneficial in various situations. Successful emotion regulation relies on a flexible choice of strategies that are suitable in given contexts, including intensity of emotions or long-term goals (Sheppes et al., 2014), and so apart from learning about the various beneficial strategies available, it would be important for individuals to also learn which strategies may be more suitable depending on what they are doing, what emotions are involved, how emotionally intensive memories are, and whether blends of strategies may be effective (Gross, 2015).

In addition, people may hold misconceptions about various strategies, which may prevent them from wanting to use them. Individuals’ beliefs about the consequences of emotion-regulation strategies are associated with their use (Ortner et al., 2017). Mamat and Anderson (2023) found that 67% of their participants saw realising the benefits of suppressing their fearful future thoughts as the biggest discovery of the suppression training that was provided to them, and 73% were surprised at their own ability to suppress. Research should further investigate what beliefs individuals hold about various emotional memory control strategies, and what training programs could have a useful impact on their memory control.

### Implementation stage of emotion regulation

After selecting a potentially beneficial strategy, an individual would then move to the *implementation stage* of emotion regulation and apply a strategy as a specific tactic. As discussed in earlier sections, some strategies of memory control may be harder to implement than some external-situation ER strategies; for example, internal attentional shifts may be harder than external gaze shifting. Suppression may be especially difficult for some individuals, as shown by the reviewed studies where suppressing attention to memories was not always successful even in the lab (Noreen & MacLeod, 2013; Satish et al., 2022); this is likely to be more so in everyday busy lives. However, people may get better at suppression with practice, as has also been demonstrated in the discussed research (Satish et al., 2022). Substituting unwanted memories (after training to notice them without delay) with a positive emotional memory could be less demanding (Noreen et al., 2016), especially if such memories were prepared and rehearsed in advance. In terms of reappraisals, distancing may require less effort to implement once learned because it can be applied in a similar way to all situations. In contrast, reinterpreting requires considering each unique situation differently (Powers & LaBar, 2019), meaning that attempting reinterpretation of each unique memory on the spot in everyday lives may not be feasible (*N.B.* sessions in the studies mentioned above took some uninterrupted dedicated time in a quiet lab space). One common issue with ER of both external and internal situations seems to be that a lot of studies conducted in the lab may not translate well to everyday situations in people’s busy lives where ER strategies (external and memory based) are needed. ER research would benefit from more ecologically valid experiments with strategies that can be applied on the spot in the real world, and from efforts to design training programs that will allow such strategies to be effectively practised.

## Final discussion

**Fig. 3:**
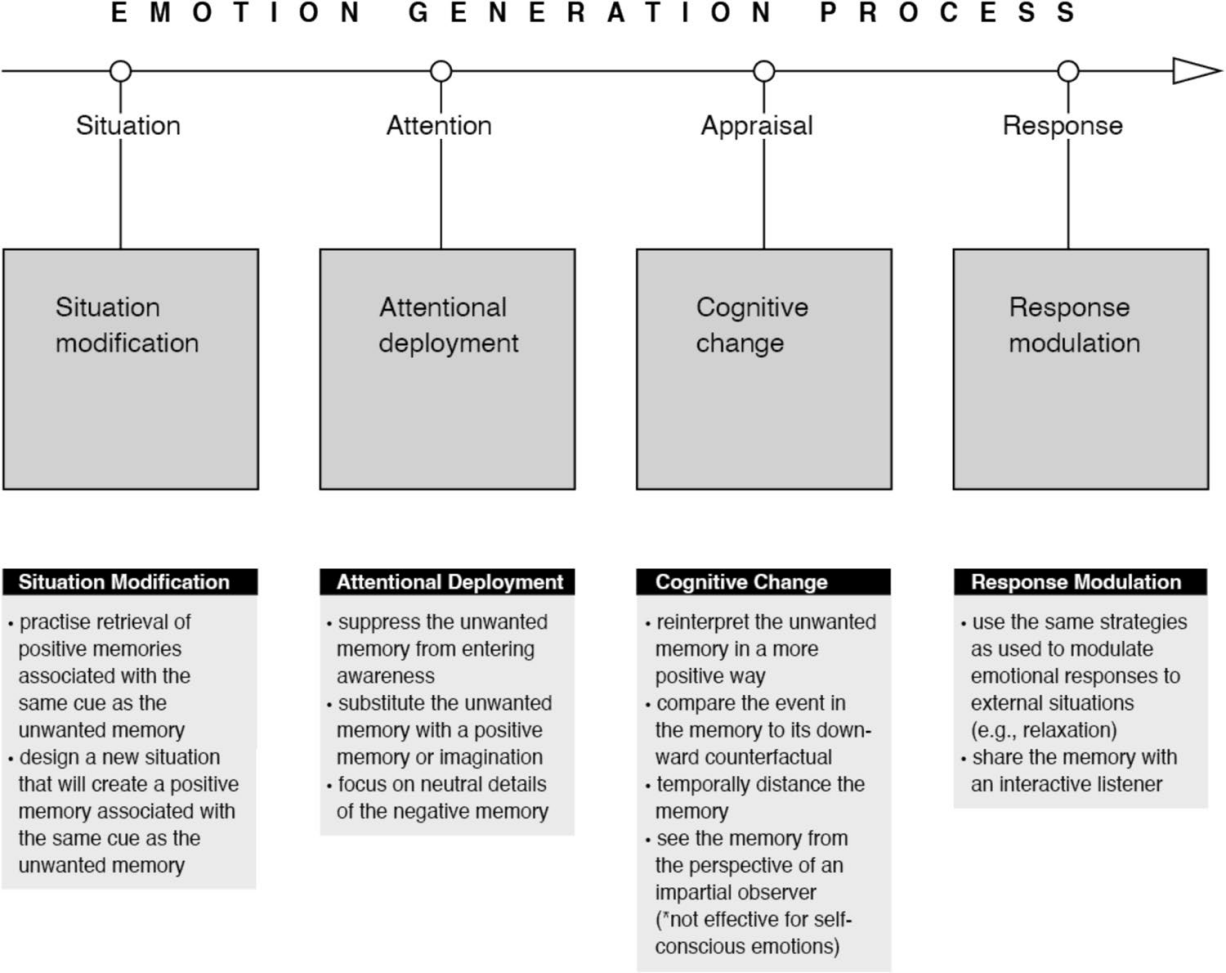
Application of Gross’s (1998a, b) Process Model of Emotion Regulation to identify memory control strategies for further research

This conceptual review has demonstrated that the Process Model of Emotion Regulation (Gross, 1998b) can be usefully applied to control of internal emotional memories, although there are some important differences compared to emotion regulation of external situations. Using the Process Model has helped to organise a number of relevant findings, identify the most potentially effective memory control strategies (see Fig. 3), and point to the most promising future research directions that may contribute to a better understanding of how people can deal with unwanted memories.

The first two stages of the model (or, in other words, the first two stages of emotion generation where emotion regulation can be applied) seem most difficult to use with memories as *situation selection* and *modification* normally refer to changes in one’s physical environment. Due to the nature of involuntary memories and how they are triggered by broad conceptual cues that are ubiquitous in our semantically rich daily lives, there does not seem to be any way to change situations in the physical environment to avoid evoking unwanted memories, apart from limited specific situations with easily identifiable triggers. Therefore, of all the stages of the model, situation selection/modification seems to be the one where strategies to control internal memories would need to differ the most from those that can be used in ER during external situations. Still, regulating emotional responses is easier and less cognitively costly in those early stages of the Process Model, before emotions fully unfold (John & Gross, 2004), and so it is worthwhile to keep investigating the possibility of memory control strategies corresponding to situation selection/modification. Therefore, we propose that instead of the physical environment, changes could be made to the internal environment of one’s associative network of cues and memories, making use of the cue-overload principle and retrieval-induced forgetting, which could involve identification and practice of positive memories associated with the same cue as the unwanted memory. This could reduce the likelihood of the most unwanted emotional memories being incidentally triggered in the future during individuals’ everyday lives. The current RIF knowledge base relies mostly on studies with memories of words and pictures, though several studies we reviewed did show that RIF can be successfully applied to autobiographical memories, too. In addition, if individuals are not able to recall positive memories associated with the cue that tends to bring up a negative memory, counterconditioning may be used to design a situation that will create such a new positive memory. Further empirical research is needed to test how such strategies can be best used to control autobiographical memories.

In the *attentional deployment* stage of the model, studies that use Directed Forgetting and Think/No-Think paradigms show that individuals are, to different extents, able to internally control attention to autobiographical memories according to their goals. Such attempts are more difficult than shifting attention to external situations (where it is possible to, e.g., avert one’s gaze) and do not always work but seem to get better with practice. As in the situational stages, most research on attentional strategies have used memories of words and pictures, offering a useful foundation, but studies with autobiographical memories are limited. Still, as shown in our review, they do offer promising findings. Specifically, suppressing an unwanted memory from entering awareness, substituting a negative memory with a positive memory or imagination, or focusing on neutral aspects of a negative memory have been shown to help control autobiographical memories by being able to stop retrieval when unwanted, reducing accessibility at delay, or improving negative emotions in response to memories. Further empirical research is necessary to better understand effectiveness of suppression, substitution, and changing focus when applied to autobiographical memories. Individual differences (such as in suppression ability) also need to be studied further in the context of autobiographical memories.

A review of studies within the *cognitive* *change* stage of the ER model showed that cognitive reappraisal of recalled emotional autobiographical memories can be very similar to cognitive reappraisal of current external situations, with the same strategies used in both contexts. Therefore, in this stage, there have been many studies that used autobiographical memories, and they showed that reappraisal can reduce negative emotional rating of reappraised memories. Different kinds of reappraisal can be effective, including reinterpretation (such as positive-meaning finding or comparing the memory to its worse-scenario counterfactual) and distancing (involving temporal distance or third-person visual viewpoint, the latter only effective for certain emotions). Most research looked only at immediate effects, and only one study found more positive emotions even 2 months after the reappraisal session, with aspects of positive reappraisals included in participants’ recollections of the original memories. More research is necessary to identify the most effective cognitive change strategies that could be applied in individuals’ daily lives; additionally, more work is needed to understand the most favourable conditions under which an autobiographical memory can be updated long term (reconsolidated after reactivation) to include aspects of a positive reappraisal as part of the memory itself.

As for the final stage of the model, *response modulation* of an already fully unfolded emotion, there is no evidence to suggest that modulation of emotions evoked by memories is different from when emotions are evoked by external situations, and, therefore, would not require a separate line of research. However, one strategy, social disclosure with an interactive listener, showed more long-term effects as it helped to increase the fading of the emotional intensity associated with an unpleasant autobiographical memory. Further research is needed to understand the types and conditions of social disclosure that may be most beneficial.

**Fig. 4:**
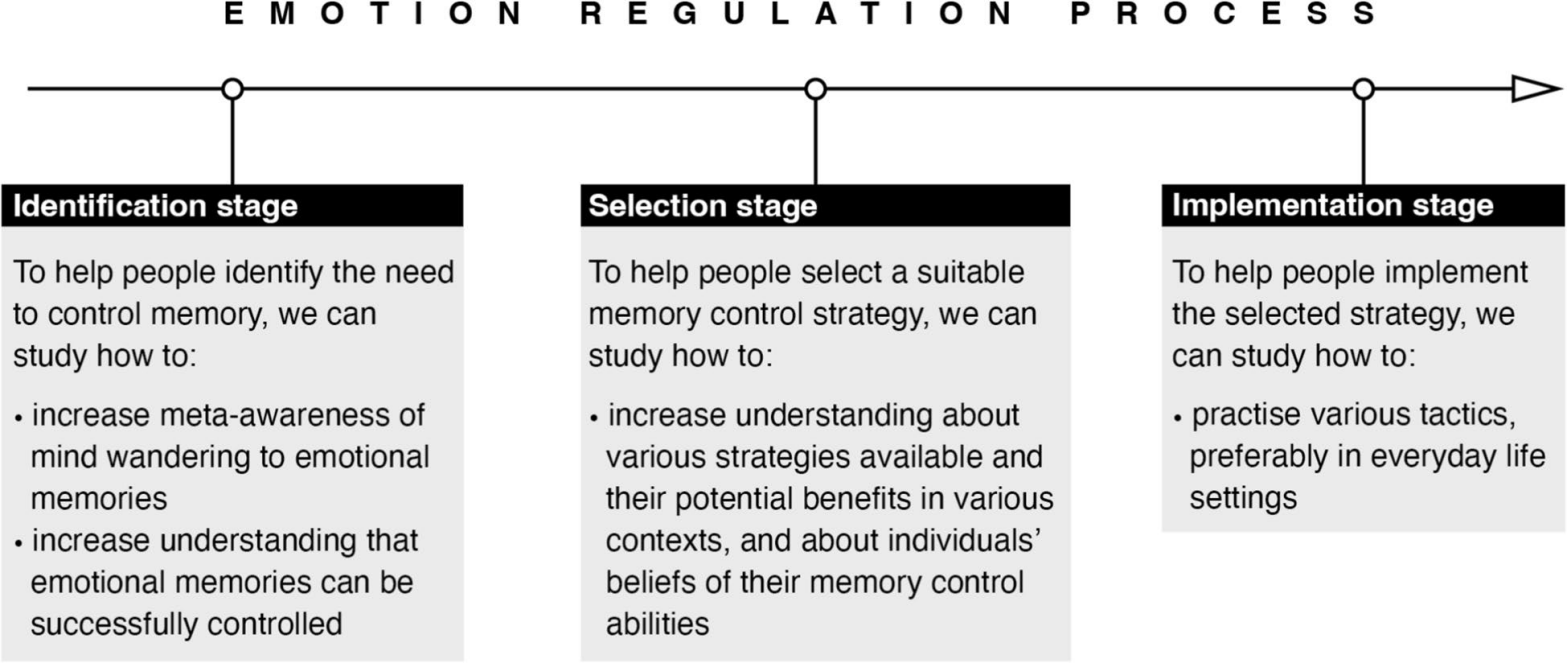
Application of Gross’s Extended Process Model of Emotion Regulation (2015) to identify further research directions in memory control

As seen in Fig. 4, in terms of the three stages of emotion regulation (as opposed to the five stages of emotion generation that can be the target of emotion regulation), research efforts could focus on ways and effects of increasing individuals’ meta-awareness of mind wandering and their understanding that memories can be controlled. Studies could also explore the effectiveness of teaching people about various strategies available for different contexts, and of clarifying individuals’ beliefs about consequences and their own abilities regarding various strategies. More research is also needed on the best ways strategies can be practised, with a focus on those that can be implemented in people’s busy everyday lives.

All the strategies identified in this review would require further empirical research to test and evaluate various protocols. Their effectiveness can be measured through either one or more of the following ways used in the reviewed research: decreased negative valence of emotions associated with unwanted memories both at the moment as well as long term, decreased emotional intensity associated with an unpleasant memory, being able to successfully stop memory retrieval when unwanted, or reduced accessibility of an unwanted memory.

The application of the Process Model of Emotion Regulation (Gross, 1998b, 2015) to emotional memories that we present here has served to enhance conceptual clarity of the field of memory control, helping to organise existing findings, revealing meaningful similarities and differences between various strategies, identifying the most potentially effective memory control strategies, and pointing to the most promising future research directions.

## Data Availability

This is a theoretical review so this is not applicable.

## Code availability

No code was used in this study.
